# Efficiency of biofilm removal by combination of water jet and cold plasma: an in*-*vitro study

**DOI:** 10.1186/s12903-022-02195-1

**Published:** 2022-05-06

**Authors:** Rutger Matthes, Lukasz Jablonowski, Vinay Pitchika, Birte Holtfreter, Christian Eberhard, Leo Seifert, Torsten Gerling, Laura Vilardell Scholten, Rabea Schlüter, Thomas Kocher

**Affiliations:** 1 Department of Restorative Dentistry, Periodontology, Endodontology, Preventive Dentistry and Pedodontics, Dental School, University Medicine Greifswald, Fleischmannstr. 42, 17475 Greifswald, Germany; 2 Imaging Center of the Department of Biology, University of Greifswald, Greifswald, Germany; 3 Sirona Dental Systems GmbH, Bensheim, Germany; 4 ZIK Plasmatis, Leibniz-Institute for Plasma Science and Technology e.V. (INP), Greifswald, Germany

**Keywords:** Biofilm, Water jet, Cold plasma, Titanium surface, Peri-implantitis

## Abstract

**Background:**

Peri-implantitis therapy is a major problem in implantology. Because of challenging rough implant surface and implant geometry, microorganisms can hide and survive in implant microstructures and impede debridement. We developed a new water jet (WJ) device and a new cold atmospheric pressure plasma (CAP) device to overcome these problems and investigated aspects of efficacy in vitro and safety with the aim to create the prerequisites for a clinical pilot study with these medical devices.

**Methods:**

We compared the efficiency of a single treatment with a WJ or curette and cotton swab (CC) without or with adjunctive use of CAP (WJ + CAP, CC + CAP) to remove biofilm in vitro from rough titanium discs. Treatment efficacy was evaluated by measuring turbidity up to 72 h for bacterial re-growth or spreading of osteoblast-like cells (MG-63) after 5 days with scanning electron microscopy. With respect to application safety, the WJ and CAP instruments were examined according to basic regulations for medical devices.

**Results:**

After 96 h of incubation all WJ and CC treated disks were turbid but 67% of WJ + CAP and 46% CC + CAP treated specimens were still clear. The increase in turbidity after WJ treatment was delayed by about 20 h compared to CC treatment. In combination with CAP the cell coverage significantly increased to 82% (WJ + CAP) or 72% (CC + CAP), compared to single treatment 11% (WJ) or 10% (CC).

**Conclusion:**

The newly developed water jet device effectively removes biofilm from rough titanium surfaces in vitro and, in combination with the new CAP device, biologically acceptable surfaces allow osteoblasts to grow. WJ in combination with CAP leads to cleaner surfaces than the usage of curette and cotton swabs with or without subsequent plasma treatment. Our next step will be a clinical pilot study with these new devices to assess the clinical healing process.

**Supplementary Information:**

The online version contains supplementary material available at 10.1186/s12903-022-02195-1.

## Background

Microbial biofilms of the oral cavity are highly diverse and can also attach on artificial surfaces like implants [1]. If the supragingival plaque is not removed regularly and meticulously from an implant, the microorganisms can migrate to deeper areas of the peri-implant tissue and cause peri-implantitis. Up to 45% of patients with dental implants are affected by peri-implantitis [2], which is characterised by inflammation around the implant, accompanied by loss of peri-implant bone. The inflammatory process is associated with an imbalance of oral microorganisms and host defence [3], as the embedded microorganisms cannot be eliminated, which is the main biologic cause for long-term implant failure [4]. Other causes such as individual bone quality, smoking, diabetes, implant design or particles released from the implants play a minor role [5], that underlines the importance of cleaning and biofilm removal during peri-implantitis treatment.

These aspects make it clear that peri-implantitis therapy represents a major challenge for the dentists. The reasons therefore are, that the microbial adhesion and the complex biofilm growth along the screw topology and the micro-rough implant surface protect microorganisms against host defence and require an effective, elaborate biofilm debridement [6–8]. Different mechanical treatments are currently used for implant debridement [9, 10]. However, mechanical procedures do not remove the complete biofilm and can damage the micro-rough structure of the implant surface [10, 11]. Furthermore, a combination of mechanical debridement with additional antiseptic treatment leads to unsatisfactory results [12, 13] and the evidence for using local antibiotics is lacking [13]. Probably, incomplete biofilm removal is responsible for the unpredictable healing. Therefore, an effective but non-destructive debridement method is necessary to improve the success rate of peri-implantitis therapy. An alternative treatment method by using a mixture of air, water and organic particles, an effective non-destructive biofilm removal was demonstrated in-vitro for air-polishing on rough titanium surfaces in a previous study [14]. However, these devices are not intended for surgical interventions, because both media—air and water—and respective tubing are not sterile. The air flow of the devices may also cause emphysema [15, 16].

To overcome the problem of insufficient instrumentation and the concomitant loss of hydrophilicity [17] we developed a new treatment system, a new handpiece for an existing water jet device and a new cold atmospheric pressure plasma (CAP) device, with enhanced ergonomic access both validated for surgical application. The CAP is suited to functionalise the water jet treated implant surface to support cell adherence and osseointegration [18]. Both devices are adapted for use in the mouth.

The water jet mechanically removes the biofilm and CAP renders surfaces hydrophilic. Early studies showed that a high-pressure pulsating water jet was more effective in reducing the bacterial load and removing debris from contaminated war wounds than low-pressure methods, like syringes [19–21]. Nowadays pressurised lavage with powered devices is used to decontaminate infected skin wounds before and after grafting, ulcers, explosion injuries, or wounds with healing disorders [22, 23]. Within the dental field, the interest in using pressured water for biofilm removal increases presently [24].

The reactive compounds of CAP, mainly reactive oxygen and nitrogen species within the gas stream, generated by the excited atoms and molecules in contact with the surrounding air, can increase the wettability of the titanium, which improves early cell attachment and early healing [25–30]. Additionally, CAP inactivates microbial residues due to its physical properties. Oxidative reactive species carried by a gas could reach hidden microbes in these niches and inactivate the microorganisms [31–33].

In this study, a newly developed cold atmospheric pressure plasma jet (CAP), called periINPlas, was applied to microbially contaminated titanium surfaces after mechanical treatment with the water jet (WJ) or with a curette and cotton swab (CC). CAP alone was not tested because previous studies have already shown that biofilm cannot be removed with a plasma jet [14]. After treatment, the decontamination was evaluated by measurement of turbidity of supernatant for microbial re-growth, and by scanning electron micrographs to analyse osteoblast-like cell spreading on the treated surface. Following hypotheses were investigated: (1) WJ removes more biofilm than CC, (2) the combined treatment with WJ and CAP results in better microbial decontamination than CC + CAP, (3) additional CAP application leads to “biologically acceptable” implant surfaces and allows osteoblast-like cell spreading after the seeding. The null hypothesis stated that there is no difference in cleaning efficacy between the water jet and the cotton swab treatment, and no additional benefit by using CAP. The discs were termed “biologically acceptable” if a disc is sterile after treatment, and osteoblastic cells could spread on the treated surface. Additionally, we tested safety and risks according to ISO/IEC standards of these devices.

## Methods

### The water jet device, assessment of the basic application risk

**Fig. 1:**
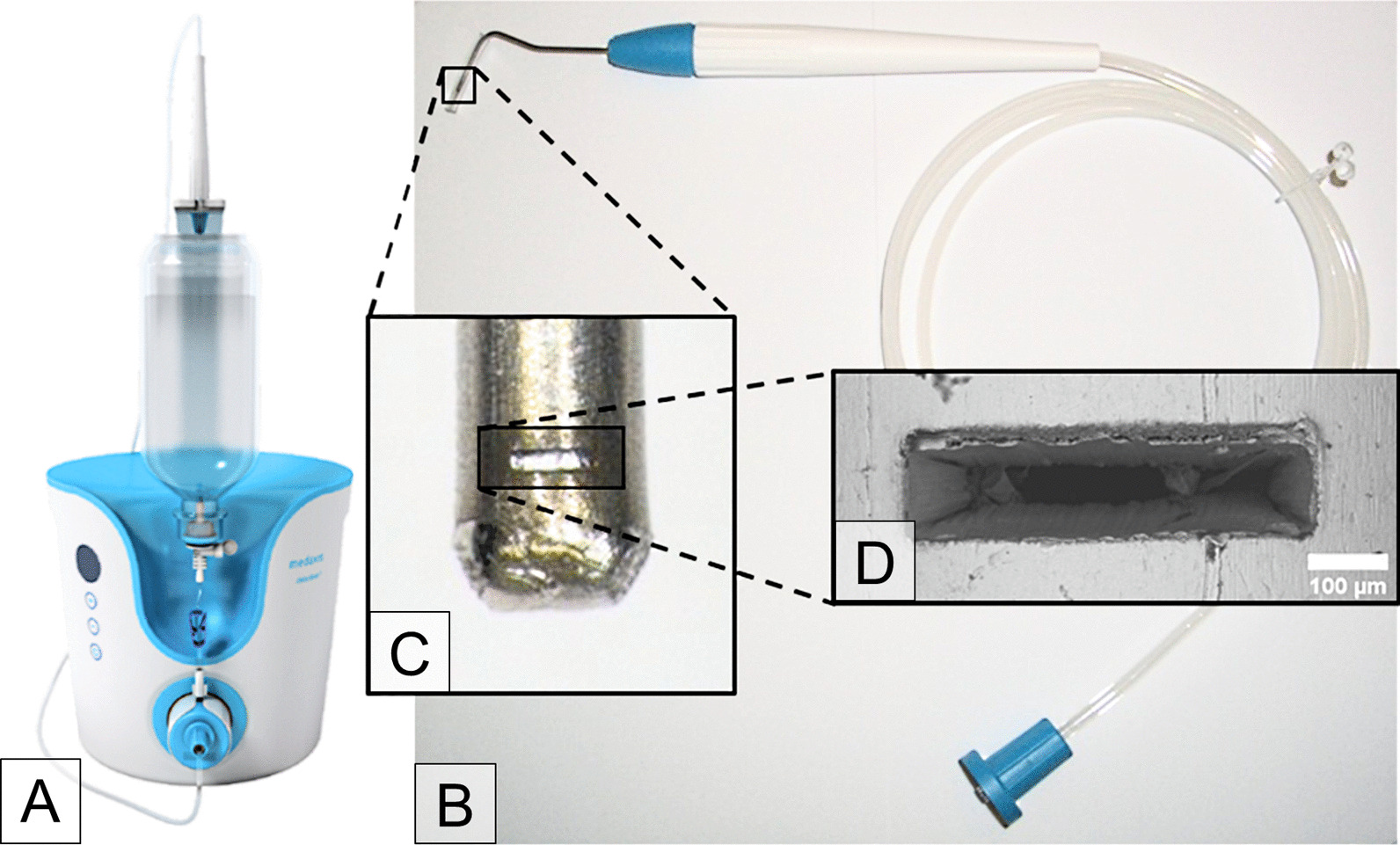
The photograph shows **A** the debritom + as manufactured by the Swiss company Medaxis AG. Our **B** adapted handpiece has an elongated application tip resembling a periodontal probe. The nozzle at the end of the tip, see a photograph in the inset **C**, produces the micro water jet for cleaning of dental implants. The nozzle has a size of about 500 × 100 µm, as can be seen in the scanning electron micrograph **D**

The Dental water jet is based on the debritom + manufactured by the Swiss company Medaxis AG, see Fig. 1A. The debritom + is CE-certified for cleaning, irrigating and debriding wounds and other diseases of the skin by using micro water jet technology.

We developed a new application tip and nozzle for the debritom + handpiece to allow its use in the oral cavity, especially the insertion of the tip into the bony defect around the implant being treated (Fig. 1B). The application tip in the form of a periodontal probe is made from a stainless-steel tube with an outer/inner diameter of 1.2/0.6 mm (Fig. 1C, D). At the end of the tip is a rectangular nozzle that is microfabricated using a short-pulse laser in a newly developed manufacturing process. The nozzle produces a micro water jet with a cross section increasing with the distance from the nozzle (Sirona Dental Systems, Bensheim, Germany). This allows efficient cleaning by mechanical means in the immediate vicinity of the nozzle but reduces jet push pressure to a safe level for distances > 1 mm. Sterile physiological saline solution (PSS, 0.9%, Fresenius Kabi, Bad Homburg, Germany) was used as the jet medium. The tests were done at intensity level 3 of debritom + , corresponding to a flow rate of 72 ± 2 ml/min. All accessories of the device, like PSS, pump, tubing and handpiece, are provided sterile. This allows the Dental water jet to be used for surgical procedures in accordance with the guidelines of the Robert Koch Institute in contrast to air-polishing devices.

Risk management of medical devices was performed according to ISO 14971 [34]. To demonstrate conformity to Essential Requirements of European Medical Device Directive which is a prerequisite for clinical testing of a new device, safety was evaluated according to IEC 60601-1 [35], electromagnetic compatibility according to IEC 60601-1-2 [36] and biological safety according to ISO 10993 series. The device was also developed in accordance with IEC 62366-1 [37] to ensure adequate usability. This study proves the biological effectiveness of the Dental water jet.

Potential side effects of WJ were evaluated in in-vitro experiments in comparison to a marketed dental air polishing device on mucosal tissue of pig jaws. The keratinized and non-keratinzed oral mucosa was treated directly with maximal 1 mm distance with water pressure intensities from level 1 to 5 (n = 4). As comparator to WJ we used the powder-water air polishing device (AIRFLOW Master Piezon®, EMS, Nyon, Switzerland) with glycine powder (particle size 25 mm, connected to the dental unit (air pressure 4.75 bar, water pressure 2.5 bar).

### Cold atmospheric pressure plasma device, assessment of the basic application risk

**Fig. 2:**
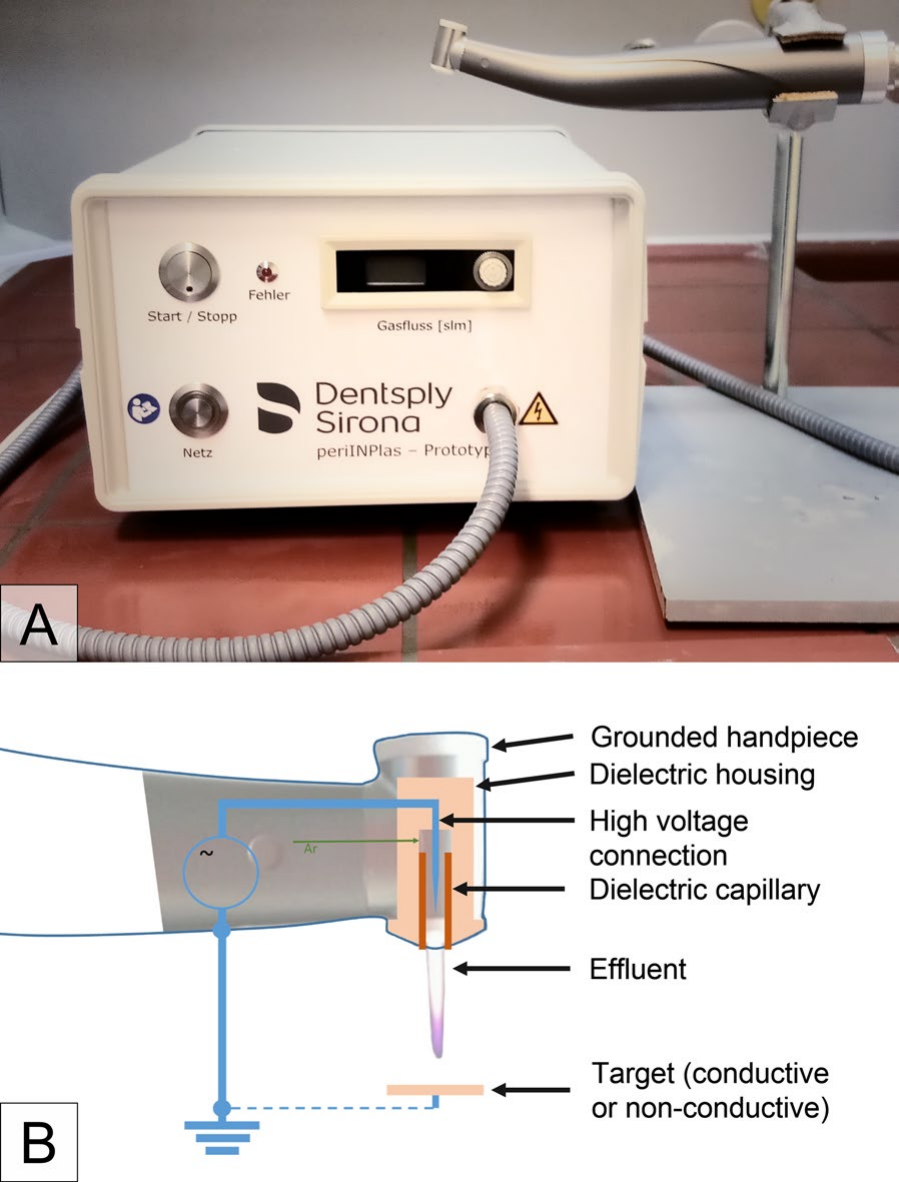
**A** Photograph of the plasma device periINPlas with handpiece, **B** schematic construction inside the handpiece head where the plasma is generated

The new system called periINPlas, developed by the Leibniz Institute for Plasma Science and Technology (INP), Greifswald, Germany, is an argon-based plasma jet with enhanced ergonomic properties due to the incorporation of plasma technology into the sleeve and head of a commercially available dental contra-angle handpiece—the T1 Classic handpiece (Sirona Dental Systems GmbH) (Fig. 2). It worked at a frequency of 0.95 MHz at 2–3 kVpp and 1.6 W maximal input DC-power. The noble gas argon (99,999%, ALPHAGAZ, Air Liquide, Düsseldorf, Germany) was used as carrier gas at a flow of 2.3 slm (standard litre per minute) controlled by an additional flow controller (MKS Instruments, Munich, Germany).

As part of the risk management according to ISO 14971 [34] and in order to qualify this newly developed device according to an upcoming standard in plasma medicine, the device was subjected to a complete test of safety conditions according to DIN SPEC 91315 [38, 39]. To further qualify the device for a multicentric clinical pilot study, reproductions of the device underwent a set of measurements to qualify reproducibility of the new system.

The temperature of the plasma source was measured at a distance from 0 to 15 mm towards the capillary edge. A fiber optic temperature sensor was positioned end-on with an x–y-z manual linear stage system for maximal signal intensity (FOT Labor Kit, LumaSense Technologies, Inc. GmbH, USA). For optical emission spectroscopy (OES) in the ultraviolet (UV), visible (VIS), and near infrared (NIR) region a calibrated fiber optic spectrometer (AvaSpec-3648-USB2, Avantes, Apeldoorn, Netherlands) was used. The system was placed end-on and connected via an optical fiber shielded by a quartz plate to measure the emission spectra. The acquired spectra were afterwards spectrally weighted according to DIN SPEC 91315 [38, 39] and the maximal exposure limit extracted. To determine the patient leakage current (PLC), a RC-circuit according to IEC 60601-1 via UNIMET®800ST (Bender GmbH & Co. KG, Germany) was connected to a copper surface, which was placed in front of the device on a one-dimensional linear stage.

The CAP device has also been evaluated for safety according to IEC 60601-1 [35], electromagnetic compatibility according to IEC 60601-1-2 [36] and biological safety according to the ISO 10993 series [40, 41]. Development was in accordance with IEC 62366-1 [37] to ensure adequate usability.

### Titanium discs

For experiments, sand-blasted, acid-etched sterile titanium discs (Dentsply Sirona Implants, Hanau, Germany) with a diameter of 6 mm, thickness of 2 mm, and Ra = 723 ± 123 nm (measured by Dektak 3 St Surface Profilometer, Irvine, CA, USA) were used.

### Cultivation of biofilms

Subgingival plaque was collected with curettes from deep pockets of the same periodontally diseased volunteer for each of the two experimental runs and was placed in tubes with culture media Schaedler Broth (Carl Roth, Karlsruhe, Germany), and incubated for 24 h at 37 °C / 5% CO_2_ to serve as inoculum for biofilm cultivation. Plaque removal was approved by the ethics committee of the University Medicine Greifswald (Registration number: BB 094/19). Titanium discs were placed into wells in a 96-well micro-titre plate (Techno Plastic Products AG, Trasadingen, Switzerland), covered with 100 µl subgingival human plaque suspension, and cultivated in broth for 7 d. The medium was replaced every 24 h. After cultivation, the medium was removed, and the biofilm-covered discs were transferred into new wells of a sterile micro-titre plate for treatment.

### Test groups

**Fig. 3:**
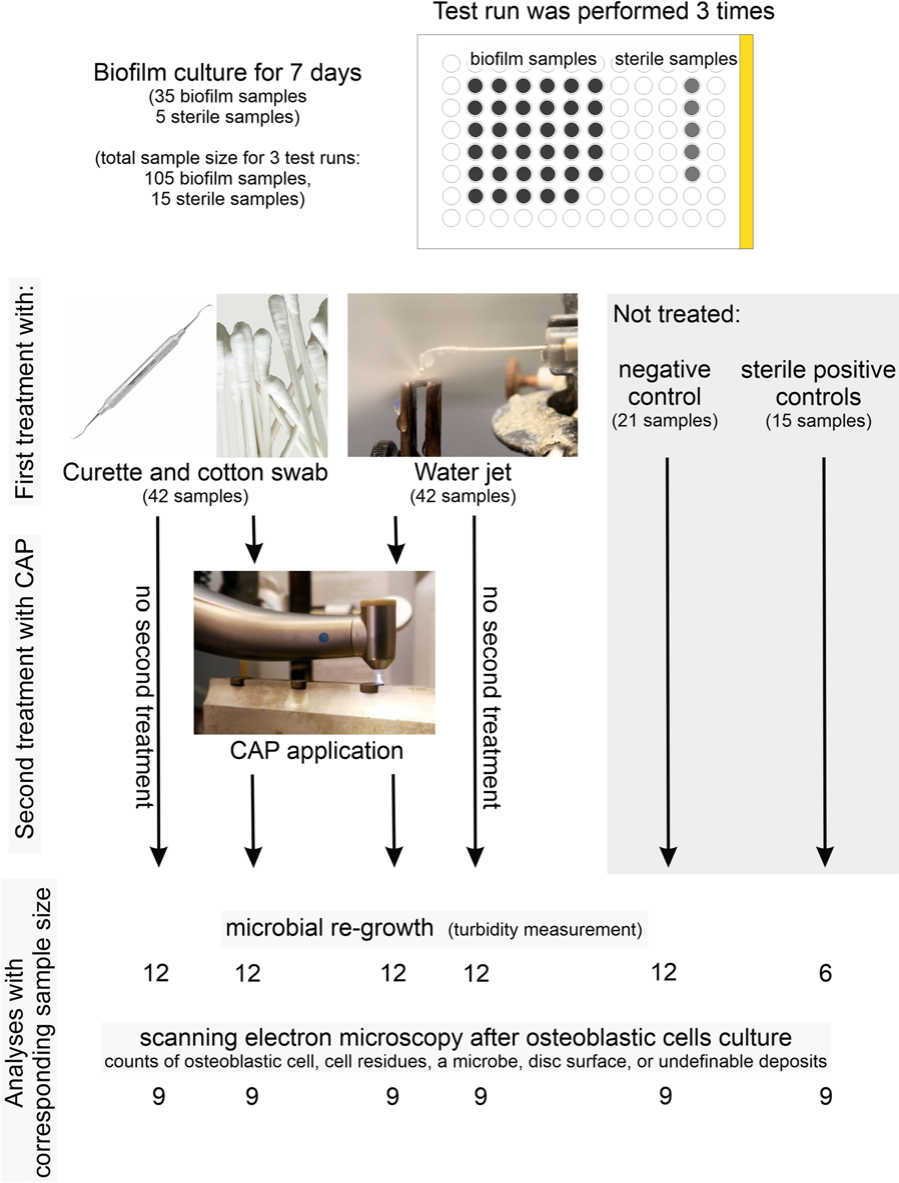
Flow chart of the timing (1st period biofilm culture for 7 days in the 96-well microplate), the distribution of the discs and number of samples among the test groups (curette and cotton swab, water jet and cold atmospheric pressure plasma (CAP) and among the analytical methods

The biofilm covered discs served as a basis for the following four test or control groups: curette and cotton swab (CC), water jet (WJ), and the combination of mechanical and cold atmospheric pressure plasma (CAP) treatment CC + CAP, and WJ + CAP; untreated discs without biofilm cultivation were used as a positive control (PC), and untreated biofilm covered discs were used as a negative control (NC). A flow chart of the test procedure and the distribution of the discs is shown in Fig. 3.

### Curette and cotton swab treatment (CC)

The discs were fixed in the disc holder for the mechanical treatment with a stainless-steel curette and cotton swab as a standard method. The discs were instrumented with gentle curette strokes for 30 s, then rinsed with 1 ml sterile physiological saline solution (PSS, 0.9%, Fresenius Kabi, Bad Homburg, Germany) and subsequently rubbed with a PSS-soaked cotton swab in horizontal and vertical as well circular motions for further 30 s and finally rinsed with 1 ml PSS. This procedure was performed on both disc sides and the edge, because the lower disc`s side was contaminated during biofilm cultivation too. After treatment, the specimens were stored in microplate wells covered with PSS for further experimental steps.

### Water jet treatment (WJ)

**Fig. 4:**
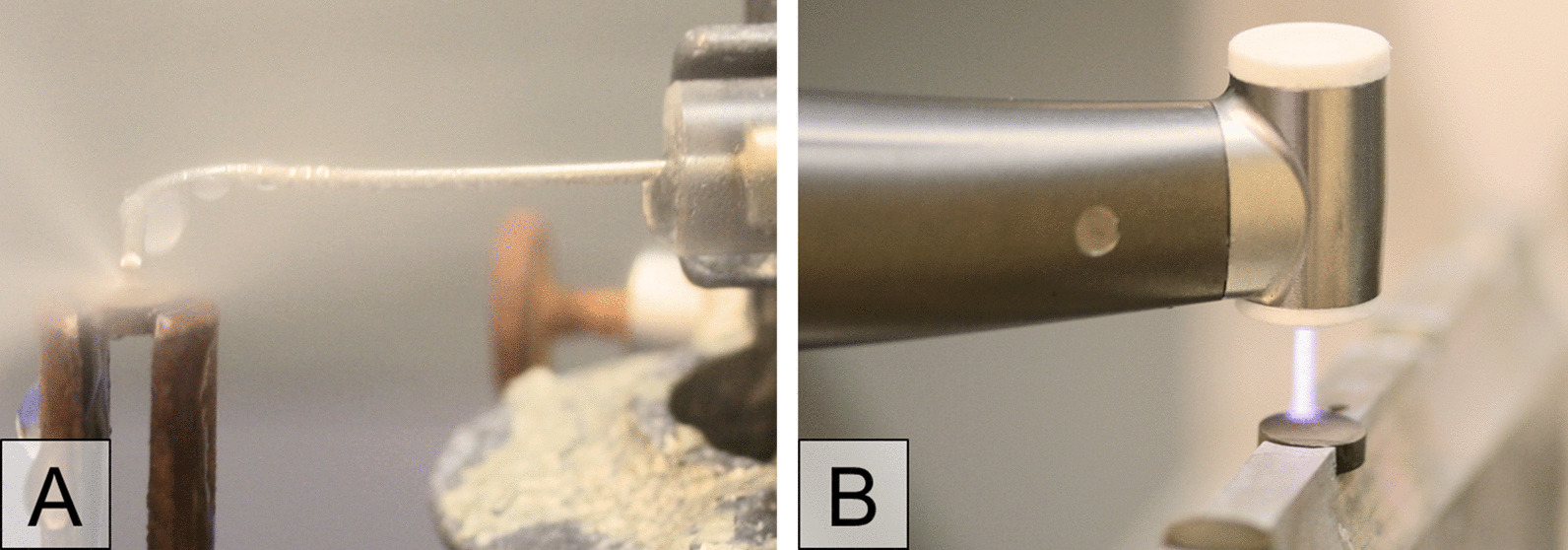
The two photographs show **A** the water jet application during treatment and **B** the plasma device handpiece during CAP treatment on titanium specimen in special disc holders

The biofilm-covered discs were fixed in a special holder and the WJ device was adjusted 0.5 mm above the discs (Fig. 4A). The specimens were treated for 30 s in a meandering pattern with the help of a slider. Both sides of the discs were treated in the described manner. The edge of the discs was treated automatically by the wide water stream during meandering movement. After treatment, the specimens were stored as described above.

### Cold atmospheric pressure plasma treatment (CAP)

A computer-controlled x/y/z stage (micos, SMC corvus eco, CA, Irvine, USA) directed the specimen holder in meandering movement 5 mm under the plasma nozzle. Specimen surface was scanned 5 × with a speed of 2 mm/s corresponding to 70 s treatment time for the total surface of one disc side (28.3 mm^2^) (Fig. 4B). The disc edge was treated during the meandering motion through the broad plasma stream. The treatment was carried out on both disc sides under an exhaust hood.

### Analysis of the biofilm removal by microbial re-growth (turbidity measurement)

After treatment, specimens were transferred into 50-ml tubes containing 4 ml of Schaedler medium and 6–8 glass pearls (diameter 3 mm) and incubated for 96 h. Turbidity was measured by a photometer (Anthos 2020, anthos Mikrosysteme GmbH, Friesoythe, Germany) at 620 nm as optical density (OD) after 0, 8, 16, 20, 24, 48, and 96 h of incubation. Therefore, at each time point, the tubes were shaken on a vortex for 20 s (the added glass pearls helped to remove coarsely adhered biofilm from the surface), and afterwards 200 µl were transferred into wells of a 96-well-microplate for measurement. Before comparing the OD values of the groups, the mean blank value of the untreated sterile specimen was subtracted from the values of all groups. To facilitate comparison across time points and groups for interpretation and graphical presentation, the OD was categorised into three scores: clear medium (OD < 0.072), slightly turbid (OD 0.072–0.220), and strongly turbid (OD > 0.220).

For microbial regrowth, each treatment modality was performed with 4 samples in parallel and replicated 3 times (n = 12 discs).

We assumed, that the longer the turbidity was delayed, the better the decontamination was. We judged discs as microbially “decontaminated” if no turbidity was detected after 4 days.

### Analysis of biofilm removal by scanning electron microscopy after culture with osteoblastic cells

For test preparation, osteoblast-like cells (osteosarcoma cells, MG-63; ATCC® CRL-1427™; LGC Standards, Wesel, Germany; RRID:CVCL_0426) were cultured in DMEM (Dulbecco's Modified Eagle's Medium Gibco, contains 2 mM L-glutamine, 1000 mg/L glucose; Thermo Fisher Scientific, MA, USA) with 10% FBS (fetal bovine serum; Gibco, Thermo Fisher Scientific, MA, USA) without any antibiotics in tissue culture flasks (TPP, Trasadingen, Switzerland). Cells were split at approximately 80% of confluence by trypsin/EDTA (PAA Laboratories, Germany) to attain an adequate number of cells for the test and further microscopic analysis of the cell surface covering. Cells were used within passages 140–151 (for origin).

After biofilm removal treatment, the cells were seeded onto the top of the discs with a density of 5.4 × 10^3^ cells /ml, which corresponds to 190 cells per mm^2^. The cells were cultured at 37 °C in a humidified atmosphere with 5% CO_2_ for 5 days.

After incubation, the samples were fixed with 2.5% glutaraldehyde in phosphate-buffered saline (PBS) and stored at 4 °C until further processing. Samples were washed three times with PBS for 5 min each, treated with 1% osmium tetroxide in PBS for 1 h, washed two times in deionised water for 5 min each, and then dehydrated in a graded series of aqueous ethanol solutions (10%, 30%, 50%, 70%, 90%) and in 100% ethanol on ice for 15 min each step. The samples were then allowed to reach room temperature before the ethanol was replaced with new 100% ethanol at room temperature for 10 min. Subsequently, samples were critical point-dried with liquid CO_2_. Finally, samples were mounted on aluminium stubs, sputtered with gold/palladium, and examined with a scanning electron microscope (SEM) EVO LS10 (Carl Zeiss Microscopy Deutschland GmbH, Oberkochen, Germany). All micrographs were edited by using Adobe Photoshop CS6.

**Fig. 5:**
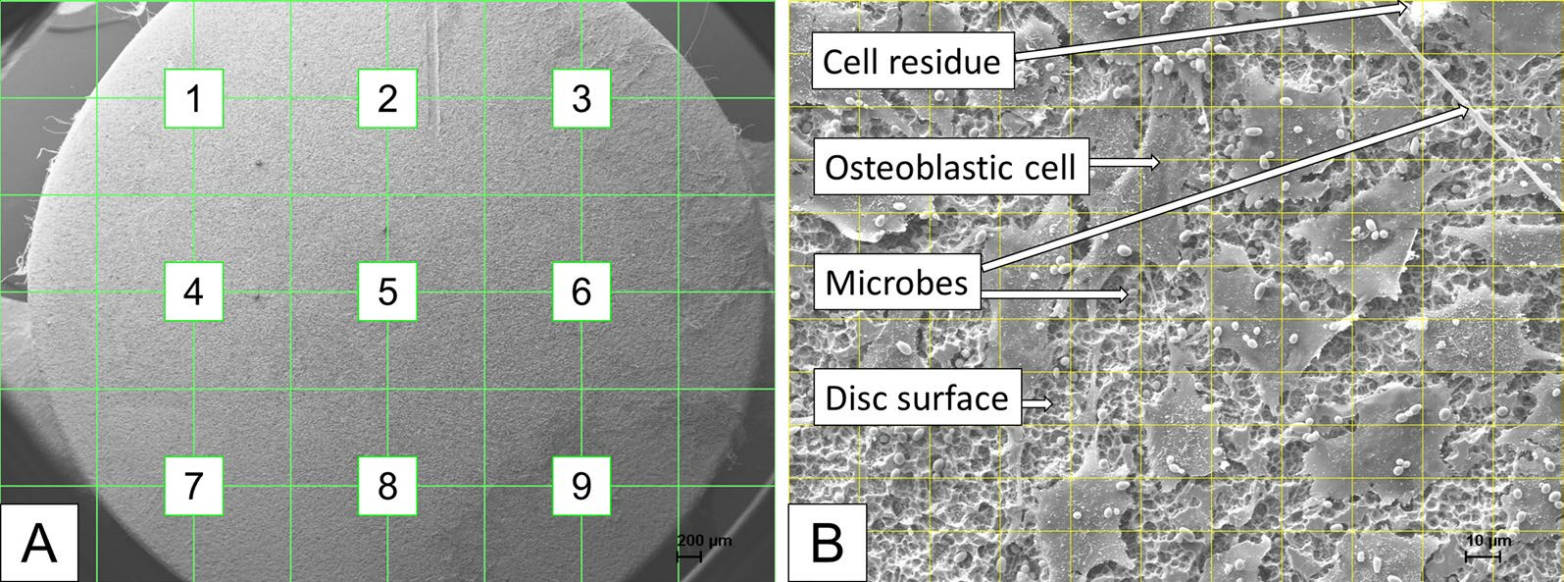
Depiction of SEM analyse procedure.** A**) Scanning electron micrograph (× 18) with a grid to define the 9 places to scan. **B** Scanning electron micrograph (× 500) with grid to count the visibility of osteoblastic cells, cell residues, microbes, blank disk surface, and objects that are not definable (deposits)

To avoid bias of cell analysis a grid with 9 intersections was applied to the image of the entire disc (magnification 18x) with the SEM software to select 9 pre-specified crossing points (Fig. 5A), at which a micrograph (magnification 500x) was then generated. Thus, 9 micrographs were captured for each disc. All filenames of all micrographs were randomised (blinded) before analysing by two trained persons of the working group independently with ImageJ (v1.50, US National Institutes of Health, Bethesda, MD). For analysis, a rectangular grid was overlaid with 10 × 10 crossing points (plugin “Grid Overlay”). At each crossing point, it was evaluated whether an osteoblastic cell, cell residues, a microbe, disc surface, or undefinable deposits was present (plugin “Cell Counter”), resulting in 900 spots per specimen, and 8100 spots per test group (Fig. 5B). Results are given as the percentage of crossing points with the corresponding surface characteristic (i.e. osteoblastic cell, cell residue, etc.) to the total number of crossing points. Selected micrographs without overlaid grid were used to present the results of different treatment procedures after 5 days of cell cultivation (Fig. 10).

For analysis of cell growth, each treatment modality was performed with 3 samples simultaneously and replicated 3 times (n = 9 discs).

We used the term “biologically acceptable” if osteoblastic cells could spread across the cleaned area after 5 days of cell culture.

### Measurement of water contact angle

The contact angle was measured using the sessile drop method with ultrapure water (Surftens Automatic 1D, OEG GmbH, Frankfurt (Oder), Germany). The water droplet (0.3 µl) was placed on the air-dried specimen surface (without prior biofilm culture) of untreated positive control (PC) and after WJ, CC, CAP, WJ + CAP, CC + CAP treatment. Three samples of each test group were measured and averaged. The specimen stayed for 2 h in double-distilled water due to feasibility and logistical issues, between specimen treatment and water contact angle measurement.

### Statistical analyses

OD values and counts of osteoblast cells, microorganisms, cell residues, blank surface, and undefinable deposits on scanning electron micrographs were presented as means with standard deviations (SD) and 95% confidence intervals (CI). Comparisons were conducted with Mann–Whitney U tests. P values were corrected for multiple-testing using the Benjamini–Hochberg method and p values < 0.05 were considered statistically significant. Analyses were conducted using Stata/SE 14.2 (StataCorp. 2015 Stata Statistical Software: Release 14. College Station, TX: StataCorp LP).

## Results

### Safety aspects of water jet device

**Fig. 6:**
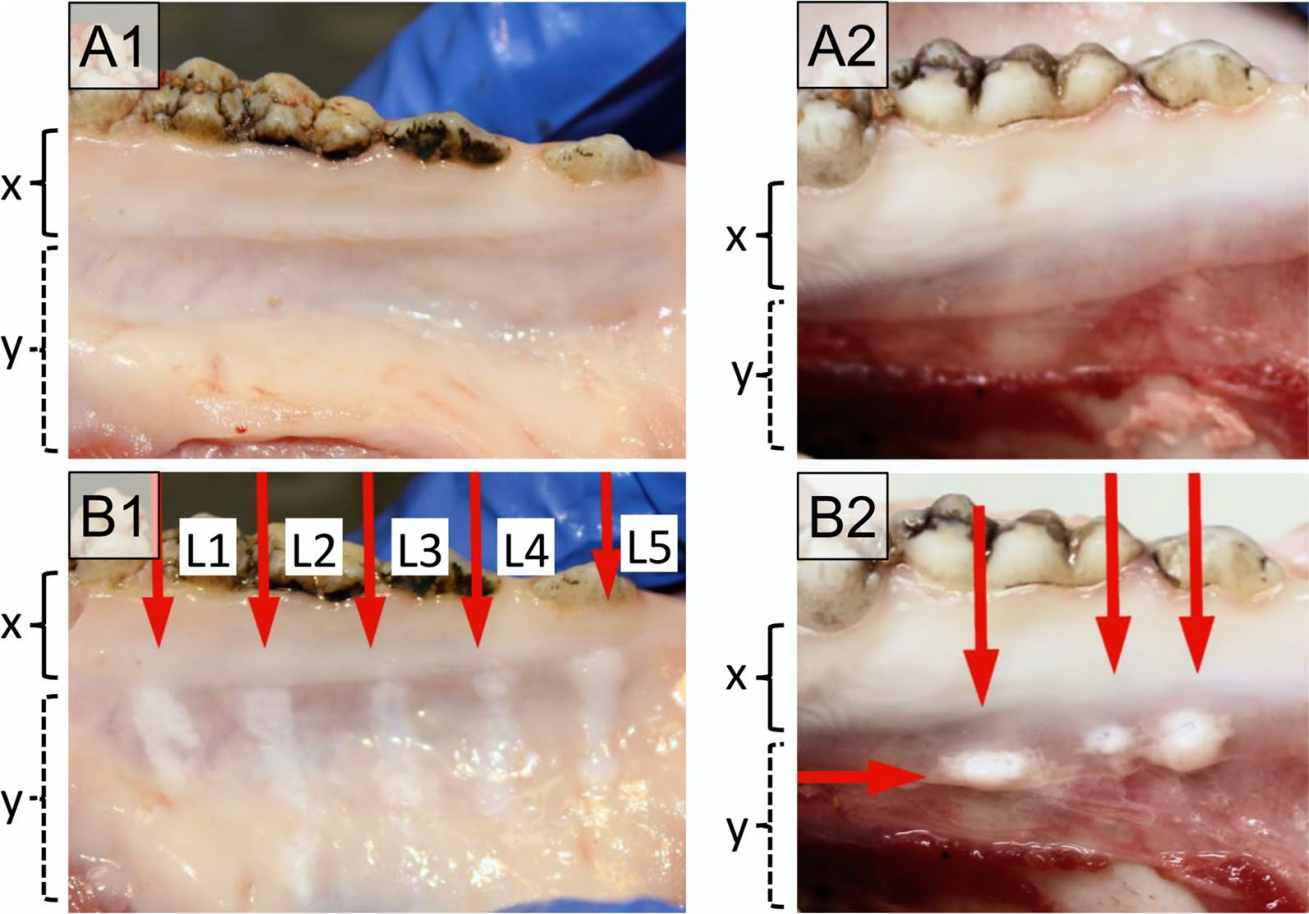
Pictures of the oral mucosa of the mandibula of pig cadavers were treated with the Dental water jet (left column, A1–B1) and a powder jet device (right column, A2–B2). Upper panel shows the tissue before (**A**) and the lower panel after treatment (**B**) in the region of keratinised gingiva tissue (x) and non-keratinised gingiva (y). The Dental water jet was used with 5 different power settings (L1–L5). Three different areas were tested for the powder jet device. No abrasion is seen in the keratinised mucosa near the teeth (x-range), tissue abrasion and blistering are observed in the non-keratinised mucosa (y-range)

Macroscopic inspection of treated pig jaws revealed no abrasions within the keratinised gingiva (Fig. 6B1, x-range), whereas within the non-keratinised gingiva, abrasions could be detected irrespective of power setting (Level “L” 1–5). The mucosal aberrations caused by an approved air polishing device were comparable (Fig. 6B2).

### Safety aspects of cold atmospheric pressure plasma device

**Fig. 7:**
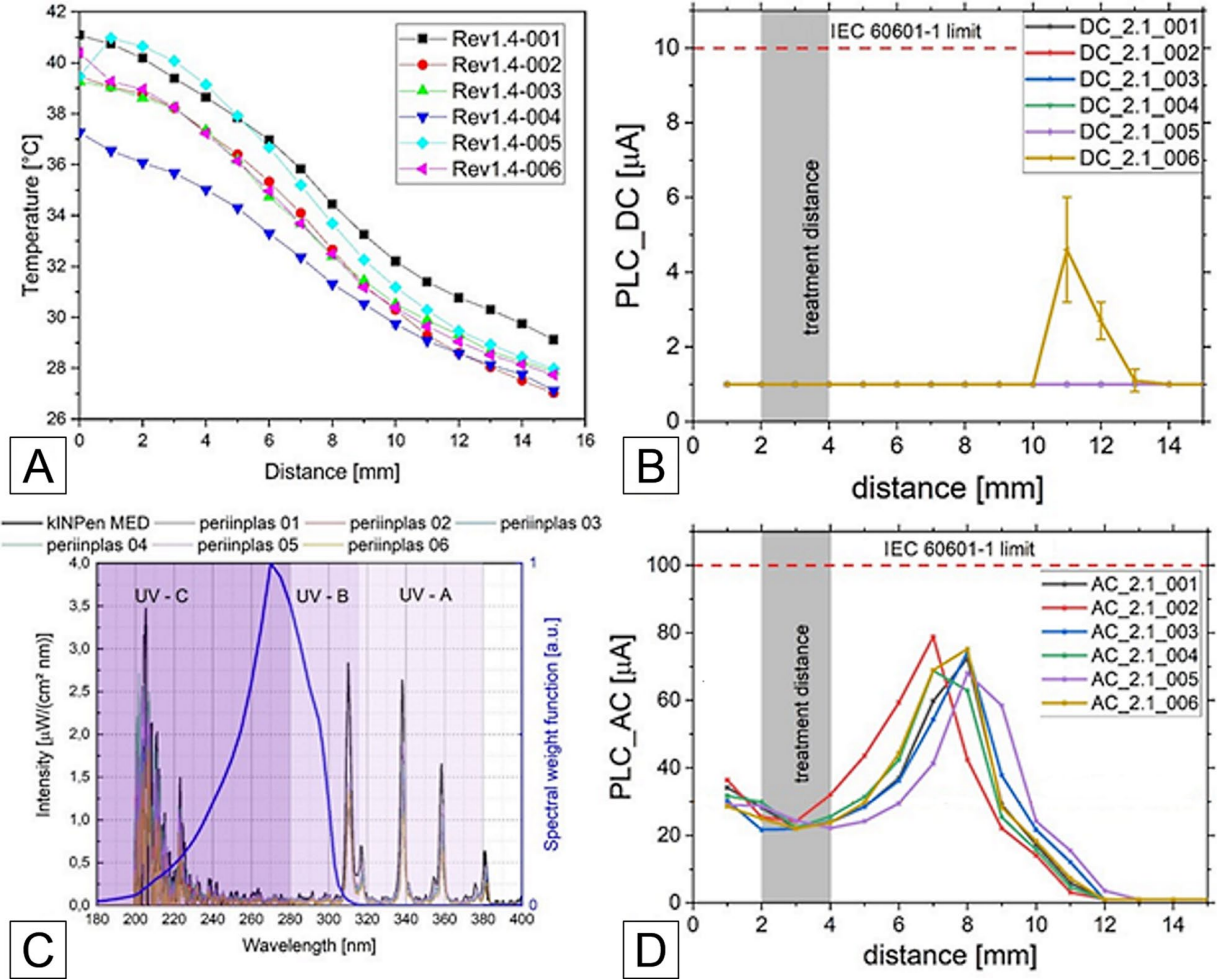
The graphs show the results of physical measurements of the different versions of the periINPlas devices, **A** temperature over distance curve, **B** DC part of patient leakage current (PLC) over distance, **C** spectral emission for all plasma devices (including kINPen MED), **D** AC part of PLC over distance

The data regarding the guideline DIN SPEC 91315 [38] presented in Fig. 7 shows the effluent temperature over distance. Considering the intended treatment distance of 2–4 mm, a temperature of 35–41 °C was measured. The DC and AC patient leakage current (PLC) values were below the limits of IEC 60601-1 [35] for all measured distances. Furthermore, the emission in the ultra-violet (UV) spectral range was far below a reference plasma jet used in medical applications, the kINPen MED [39]. The present UV irradiance resulted in a treatment limit between ten and twenty minutes per surface element depending on the used device. It must be noted, that the spectral component in the UV-C range dominated this estimation and was strongly affected by noise amplification due to the calibration process.

### Safety aspects of both devices

Both devices were tested for safe handling, risk management, electrical safety, biocompatibility and biological risks according to ISO/IEC standards, respectively according to the German and European versions as DIN/EN/ISO 14971, 60601, and 10993, and 62366 [34–37, 40, 41]. The (test) reports according to these standards were submitted to the Federal Institute for Drugs and Medical Devices for approval of the clinical investigation with this device and to the ethics committees involved for ethical evaluation. The submissions were approved (Additional file 1) which confirms the safe use of the devices to treat implants during a surgical peri-implantitis therapy in a pilot study. The pilot study is registered in the German Clinical Trials Register (DRKS00026673).

### Water contact angle measurement

**Fig. 8:**
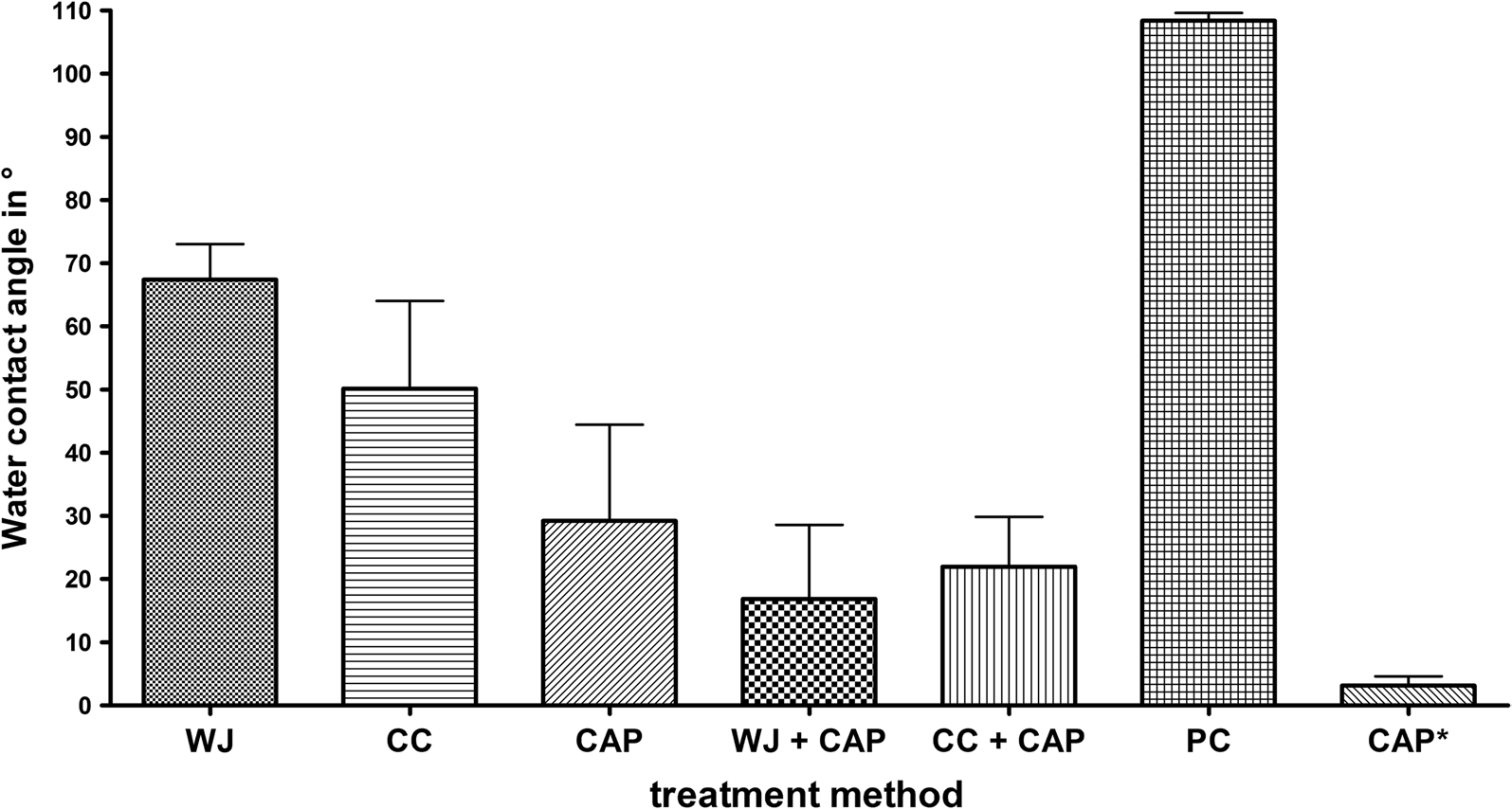
Mean values and standard deviation of water contact angles (°) of specimen treated by water jet (WJ), curette + cotton swab (CC), cold atmospheric pressure plasma (CAP), combined treatment of WJ + CAP and CC + CAP, and the untreated positive control (PC) (n = 3, time span between treatment and measurement was 2 h). Additionally, mean water contact angle data for specimen immediately measured after CAP (CAP*, time span 10 min) are shown

A reduced water contact angle was measured after all treatment procedures compared to the untreated positive control (PC), which exhibited an average water contact angle of 108°. The application of CAP resulted in a super-hydrophilic contact angle (3°, range 2°–5°), if measured immediately after treatment. It increased to 29° (range 20°–47°) after 2 h. The contact angle (after 2 h) on WJ (68°) or CC (50°) treated specimen was decreased further after subsequent CAP application to 22° (WJ + CAP) or 17° (CC + CAP) (Fig. 8). The images for the contact angle measurement are available in Additional file 2.

### Biofilm regrowth after 4 d cultivation

**Fig. 9:**
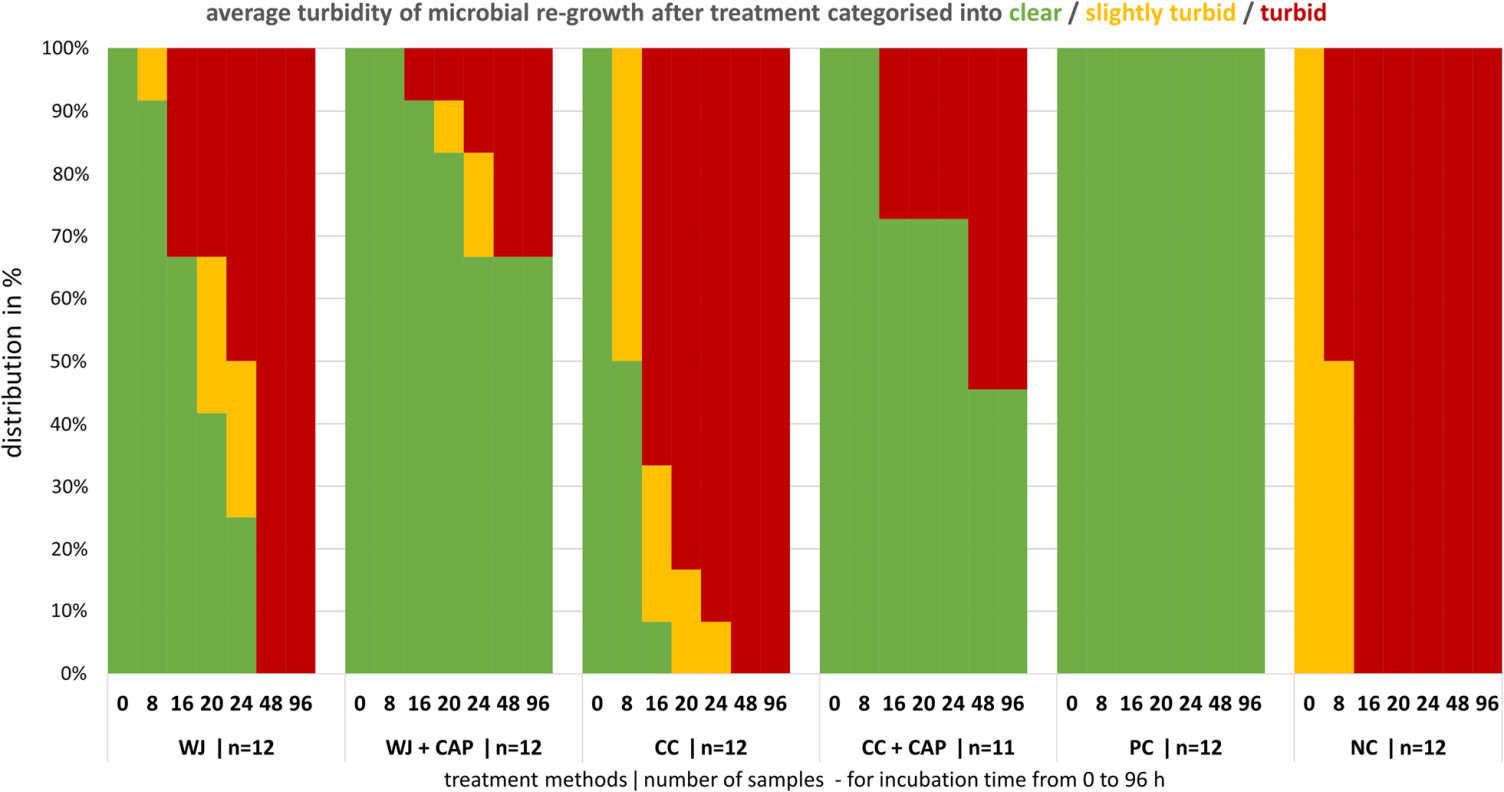
The stack diagram shows the distribution of the categorised OD values (clear, slightly turbid, and turbid) after treatment with water jet (WJ), curette + cotton swab (CC), WJ or CC combined with cold atmospheric pressure plasma CAP (WJ + CAP, CC + CAP), and the two untreated controls, the sterile positive control (PC), and biofilm covered negative control (NC). Turbidity was measured after 0, 8, 16, 20, 24, 48, and 96 h incubation time

After treatment, test specimens were cultivated at 37 °C for up to 96 h, to allow vital microorganisms to proliferate thereby increasing turbidity of the suspension. The turbidity was measured after 0, 8, 16, 20, 24, 48, and 96 h. All samples treated with CC were judged as turbid after 20 h. The same was true for WJ treated samples after 48 h. After 96 h of incubation, 67% of WJ + CAP and 46% CC + CAP treated specimens were still clear (Fig. 9).

The OD values of all treatments were significantly lower compared to the NC, until 8 h for the CC, 24 h for the WJ, and 96 h for the CC + CAP and WJ + CAP of incubation time and respective test group (Table 1). For the time points 16 h and 20 h, the turbidity differences between CC and WJ, and for the time points 24, 48 and 96 h the turbidity differences between CC + CAP and WJ + CAP treated specimen were statistically significant (Table 1).

**Table 1:** The median OD values (with 25% and 75% quantiles) of turbidity measurement after biofilm treatment and for the untreated control during incubation time until 96 h are shown (n = 12 for each test group)

Time (h)	NC	WJ	WJ + CAP	CC	CC + CAP
0	0.029 (0.024–0.034)	0* (0–0.001)	0* (0–0)	0* (0–0)	0* (0–0)
8	0.249 (0.067–0.442)	0.001* (0–0.003)	0* (0–0.001)	0.021*^,Δ^ (0.003–0.097)	0* (0–0)
16	0.595 (0.552–0.609)	0.002* (0–0.308)	0* (0–0.001)	0.326 ^†,Δ^ (0.043–0.335)	0* (0–0.312)
20	0.615 (0.571–0.641)	0.038^*^ (0.004–0.315)	0.003^*^ (0.000–0.007)	0.341 ^†,Δ^ (0.273–0.359)	0.006^*^ (0–0.328)
24	0.622 (0.545–0.640)	0.130^*^ (0.009–0.295)	0.001^*,Δ^ (0–0.034)	0.357 ^Δ^ (0.316–0.530)	0.001^*‡^ (0–0.909)
48	0.847 (0.708–0.920)	0.644 ^‡^ (0.573–0.663)	0^*,Δ^ (0–0.306)	0.692 ^Δ^ (0.552–0.747)	0.208^*‡^ (0–0.249)
96	1.242 (1.163–1.305)	1.189 ^‡^ (1.098–1.384)	0^*,Δ^ (0–0.383)	1.210 ^Δ^ (1.121–1.378)	0.252^*,‡^ (0–0.335)

*NC* negative control, *WJ* water jet, *WJ* + *CAP* water jet and cold atmospheric plasma, *CC* curette + cotton swab, *CC* curette + cotton swab and cold atmospheric plasma

Comparisons were done using Mann–Whitney U tests. Benjamini–Hochberg corrected p-values (WJ vs. CC + CAP and CC vs. WJ + CAP were not compared); *significantly different compared to NC; ^†^significantly different compared to WJ; ^‡^significantly different compared to WJ + CAP; ^Δ^significantly different compared to CC + CAP

### Cell, microbe or remnant covering after 5 days cell culture

#### Crossing points with cells

The highest counts were found on WJ + CAP treated specimen (82.0 ± 31.0%) and CC + CAP treated specimen (71.9 ± 37.5%), which was not significantly different to the PC (78.5 ± 31.4%), but to all other test groups, whereas cells were found only at 10.0 ± 26.9% on CC and 11.4 ± 29.4% on WJ treated specimen. The means of NC and of the test groups WJ and CC were not statistically different from each other (Table 2).

**Table 2:** Descriptive statistics (mean ± standard deviation) for counts of crossing points at which shown osteoblastic cells, microbes, cell residues, blank disc surface, and undefinable deposits on specimen surface were shown on images of scanning electron micrographs stratified by the treatment groups (n = 9 for each test group)

Treatment	Cells	Microbes	Cell residues	Disc surface	Deposits
PC	78.5 ± 31.4^b,e,f^	0.0 ± 0.1^b,d,e,f^	0.8 ± 1.8^b,c,d,f^	20.7 ± 30.1^d,f^	0.0 ± 0.2
NC	0 ± 0^a,c,d,e,f^	53.7 ± 31.5^a,c,d,e,f^	26.2 ± 26.2^a,c,d,e,f^	12.6 ± 18.9^d,e,f^	7.5 ± 22.1^f^
WJ	11.4 ± 29.4^a,b,c,d^	27.7 ± 33.8^a,b,c,f,d^	5.2 ± 8.9^a,b,c,f,d^	55.6 ± 39.7^a,b,c,f,d^	0.1 ± 0.7
WJ + CAP	82.0 ± 31.0^b,e,f^	0.0 ± 0.1^b,d,e,f^	0.3 ± 0.9^a,b,e,f^	17.6 ± 30.2^d,f^	0.2 ± 1.3
CC	10.0 ± 26.9^a,b,c,d^	12.2 ± 27.1^a,b,c,d,e^	2.1 ± 4.1^a,b,c,d,e^	75.7 ± 34.5^a,b,c,d,e^	0 ± 0^b^
CC + CAP	71.9 ± 37.5^b,e,f^	0.6 ± 2.8^a,b,c,e,f^	0.4 ± 1.0^b,e,f^	27.1 ± 36.0^b,e,f^	0 ± 0

*PC* positive control, *NC* negative control, *WJ* water stream, *WJ* + *CAP* water stream and cold atmospheric plasma, *CC* curette + cotton swab, *CC* curette + cotton swab and cold atmospheric plasma. Comparisons were made using Mann–Whitney U tests. Benjamini–Hochberg corrected p-values < 0.05 were considered statistically significant

^a^Statistically significant difference compared to the positive control; ^b^Statistically significant difference compared to the negative control; ^c^Statistically significant difference to the WJ + P; ^d^Statistically significant difference compared to the CC + P; ^e^Statistically significant difference compared to the WJ; ^f^Statistically significant difference compared to the CC

#### Crossing points with microbes

On CC treated discs, microbes were found at 12.2 ± 27.1% of crossing points, on WJ at 27.7 ± 33.8%, whereas on WJ + CAP (0.0 ± 0.1%) and CC + CAP (0.6 ± 2.8%) treated specimen nearly no microbes were detected. The counts of microbes were not significantly different between the WJ and CC test groups (Table 2).

#### Crossing points with remnants

Cell residues were found in every test group and increased if the number of microbes was significantly higher than the number on the PC. The undefinable deposits on surfaces of the NC were significantly higher compared to all other groups (Table 2).

### Scanning electron micrographs

**Fig. 10:**
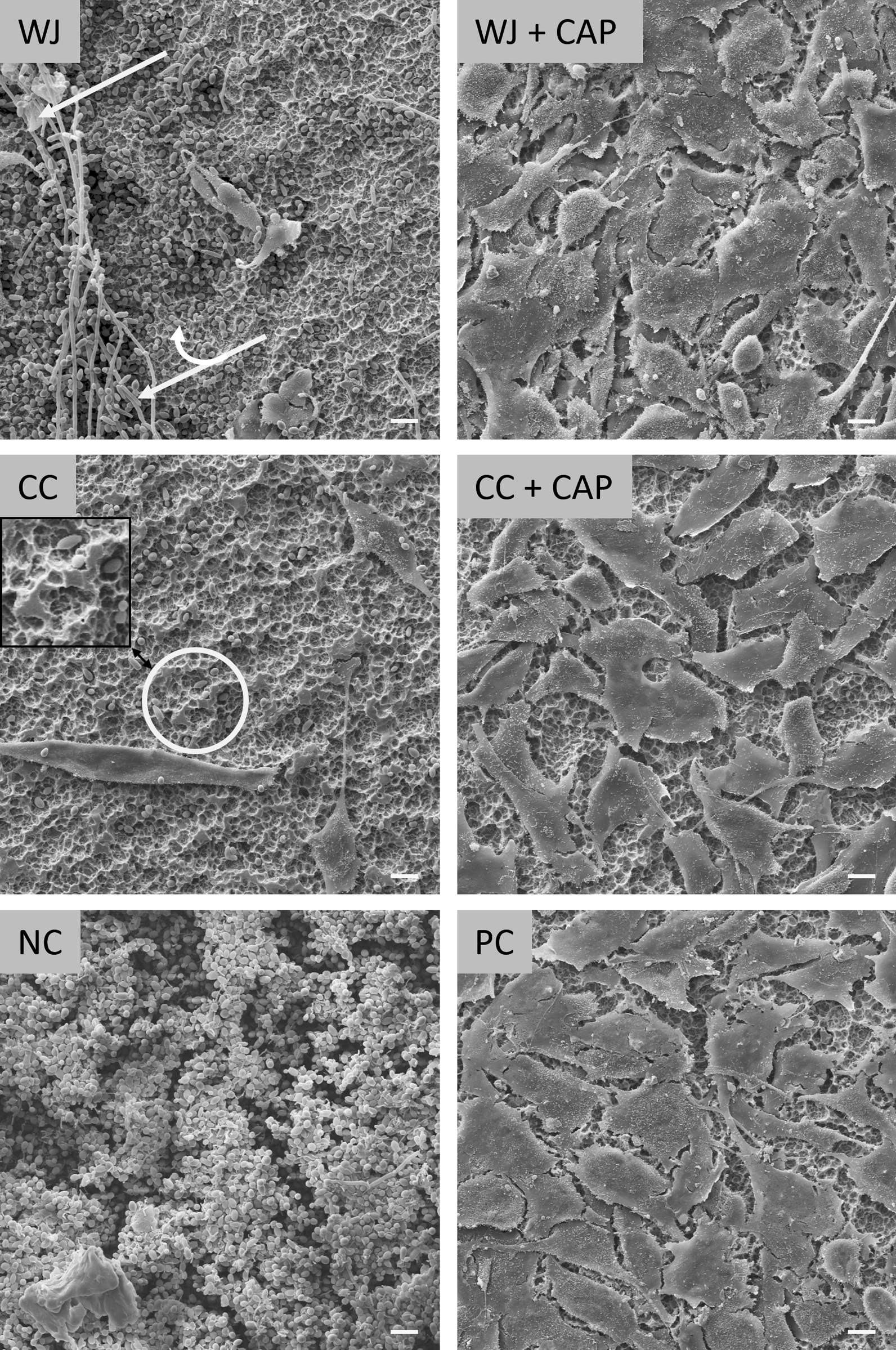
Scanning electron micrographs showing examples of specimen after 5 days of osteoblastic cell cultivation for water jet (WJ), curette + cotton swab (CC) and their combinations with plasma (WJ + CAP, CC + CAP), negative control (NC) with untreated plaque-biofilm, and untreated positive control PC. The white single arrow points to destroyed osteoblastic cells, the double arrow points to microbes, and the circle marks a scratched area (see also Additional file 3). Scale bars: 10 µm

Selected scanning electron micrographs show examples of the disc surface after 5 days of cell cultivation after treatment with WJ, CC, their combination with CAP, the controls NC, and PC (Fig. 10). NC specimens were nearly completely covered with biofilm, whereas PC displayed a nearly complete osteoblastic cell-covered surface. WJ + CAP and CC + CAP treated specimen display a similar osteoblastic cell-covered surface like PC. On both CC + CAP and WJ + CAP almost no microbes were detected, whereas on WJ and CC treated discs osteoblastic cells were destroyed or overgrown in areas with microbial regrowth (cell residues) (Fig. 10, WJ, white arrow). CC treated surfaces often displayed scratched surfaces (Fig. 10, CC, white ellipse; see Additional file 3).

## Discussion

Current surgical peri-implantitis therapy leads to uncertain success with regard to the progression or continued process of the disease [42]. Different mechanical treatment methods are available, but all available methods cannot guarantee a sufficient biofilm removal or an undamaged implant surface [43]. Follow-up treatment with supplementary bone materials or antimicrobial gels can also support the success of peri-implantitis therapy [44]. However, the key role is played by the decontamination process, which does not damage the rough implant surface structure. Therefore, the newly developed devices for the WJ and CAP treatment were investigated in this study. The WJ application tip is very thin and shaped like a periodontal probe, thus allowing access to the implant surface under constraint geometrical conditions. The nozzle at the distal end emits the water jet at an angle of 90° allowing the cleansing of the apically faced threads. That is an advantage compared to established air-polishing devices because these areas are inaccessible with air-polishing when the spray is directed at 30 to 60° degrees [45]. The cleansing efficiency of air-polishing was improved, if the device could be used perpendicular to the surface [46], which is, however, not possible in clinical practice. Furthermore, the water stream of the WJ application is broadly fanned (angle approx. 45°) so that a wide area could be treated.

The CAP device was completely redeveloped compared to previous in-vitro experiments [14], where we used the devices kINPen08, kINPen09, and kINPenMED. The old devices had a bulky handpiece and higher leakage current values at close treatment distances, which would not allow an effective or regulatory approved handling in the geometrically restricted oral cavity. For this reason, the CAP components were miniaturized to fit into a dental hand piece at a reduced gas flow range and new driving electronics. The new CAP system generates a leakage current of compliant amplitude also at very close treatment distance, while not compromising on efficacy. Furthermore, a set of CAP devices was shown to be comparable within multiple units. With our new in-vitro results we can confirm a similar antimicrobial and hydrophilicity potential and assume, that the plasma discharge properties were comparable to the formerly used plasma source kINPen09 [14] while the periINPlas CAP device successfully passed the DIN SPEC 91315 [38]. The temperature measurement of the CAP device showed a maximum temperature of 41 °C (Fig. 7A). Even if the tissue around the treated implant comes into direct contact with plasma, no temperature-related damage to human cells is expected. The determined UV radiation of the periINPlas is lower than that of the kINPen MED (Fig. 7C), a medical plasma device approved for wound treatment, so no negative effects are expected regarding UV radiation.

Due to the fact, that we misinterpreted in a previous experiment [47] scanning electron micrographs directly after treatment as clean, although microbial residues and bacteria were hidden in surface structures, we now used two different analytical approaches to verify the cleaning results. Instead of performing the usual methods of analysing biofilm removal immediately after debridement by selected microbial-stained fluorescence or scanning electron microscopy images or biofilm stains such as gentian violet or by colony forming units after ultrasonic detachment (which will not reliably detach all microbes) [48–50], we gave hidden microbes a chance to grow again. Then we determined the cleaning efficacy after a certain time lag between debridement and analysis by (1) measuring the turbidity of the growth medium up to 4 days of microbiological culture after debridement, and (2) by evaluation of the distribution of osteoblastic cells, microbes, cell residues, blank disc surface, and undefinable deposits on scanning electron micrographs across the debrided area after 5 days of cell culture [14]. Based on these two outcomes, we judged the instrumentation properties. These detection methods theoretically allow only one remaining microbe to cloud the medium or re-form microscopically detectable biofilm structures, and the combination of two detection methods improves the reliability to assess the debridement potential compared to the commonly used methods mentioned above. To ensure sterile mechanical treatment procedures, we additionally treated sterile discs with CC and WJ. Both methods (WJ, CC) showed no increase of turbidity during 4 days of culturing (Additional file 4). We concluded that our treatment procedure did not introduce any bias.

Our 1st hypothesis that WJ is superior to CC was not confirmed with both analytical methods, although microbial regrowth was somewhat retarded on WJ-treated discs compared to CC up to two days. It is unknown whether this delay has any clinical benefit. Mechanical instrumentation with saline-soaked cotton swabs, which is equivalent to our CC instrumentation, has been reported as treatment of choice in several clinical studies, but only about 45% of peri-implant cases were successfully treated [51–53].

The 2nd hypothesis that the combined treatment with WJ and CAP results in better microbial decontamination than CC + CAP could be confirmed. In addition, a superior decontamination was achieved on treated surfaces with the combined treatment (CC + CAP or WJ + CAP) compared to the single treatment with CC or WJ, which is mirrored in OD analysis and scanning electron micrographs (Table 1; Fig. 10). The highest rate of decontaminated discs was achieved after WJ + CAP and CC + CAP treatment (67% and 46% of all discs showed no microbial growth after 96 h in the turbidity measurement test, respectively), which was also reflected in superior mean osteoblastic cell count of CAP treated surfaces (82.0% and 71.9%, respectively) compared to treatment without CAP (10.0% and 11.4%, respectively). Whether the difference for the combined treatment (82.0% vs. 71.9%) results in a higher clinical benefit with WS + CAP over CC + CAP is open to debate.

The 3rd hypothesis that additional CAP treatment leads to “biologically acceptable” implant surfaces and induces increased cell spreading after cell seeding was confirmed, too. The area covered with osteoblastic cells was comparable for CC + CAP, WJ + CAP and the positive control (Table 2). These results emphasise the benefits of CAP and suggest that this combined treatment approach with CAP may overcome the present problems of instrumenting rough implant surfaces.

Previous studies of our lab used comparable methods to analyse scanning electron micrographs, which allow us to compare different treatment modalities across studies: Duske et al. investigated mechanical treatment with a plastic brush [47] and Matthes et al. with air polishing [14]. The area covered with cells was comparably low with the brush (19.7%), with CC (10.0%), WJ treatment (11.4%) but outperformed by air polishing (84.7%). However, Matthes and co-workers performed the air polishing for 90 s, whereas duration of WJ treatment was limited to 30 s, which may be one reason for the present inferior WJ results. Duration of WJ treatment was based on a preliminary study that showed that a 30-s WJ treatment was sufficient to obtain very clean surfaces analysed by standard fluorescence microscopy, which has a lower detection limit for microbial residues on the entire specimen surface than the re-growth method (data not shown). Another reason may be that air polishing uses a powder in an air–water mixture, which mechanically removes the biofilm and microbial residual fragments more efficiently than WJ. The counts of “disc surface”, as a marker for the surface area which could not be covered by cells, were very different between these studies (brush 2.8% vs. WJ 55.6% vs. air-polishing 15.2%). The reason, why cells could not spread over the surfaces cleaned with different methods cannot finally be answered here. Possibly, in the present study not identifiable microbial residues [54] or a surface that is too hydrophobic hampered cell attachment that was changed after subsequent CAP. Comparisons with other studies using only water jetting to decontaminate or clean implant surfaces are difficult, because not only the surface contamination—microbial or artificial—or surface models used, but also the physical properties (e.g. water pressure) of devices used were very different [24, 55–57].

All treatment methods decreased the water contact angle indicating an increased hydrophilicity. Increased hydrophilicity supports cell attachment and spreading [27], and thus early implant integration [26]. The untreated control disc was hydrophobic under dry conditions as indicated by a water contact angle of 108°. Cleaning by surface treatment with WJ or saline-soaked cotton swab and subsequent rinsing with saline is less efficient to decrease water contact angle compared to CAP (Fig. 8). Nevertheless, cells settled very well on PC discs (sterile and untreated discs), so in addition to hydrophilicity, an uncontaminated surface—or more specifically—a surface with low organic carbon content favours cell spreading which can be realised by CAP. To summarise, only the combined treatment approach is sufficient to generate “biologically acceptable” titanium implant surfaces, which is in conformity with previous studies [27, 30, 47].

Results of WS and air polishing treatment on pig jaws demonstrated that both devices do not induce tissue abrasion on keratinised gingiva, whereas within the non-keratinised gingiva tissue abrasion or blistering occurs (Fig. 6). With the air polishing device, in addition to water, air and powder can be introduced into the tissue, which may in rare cases cause an emphysema [15, 16, 58]. For the Dental water jet, only pure PSS is used without additional air or powder. Therefore, we expect no increased risk for emphysema, but water may be pressed into the connective tissue. More risks requiring laboratory tests were identified in the risk management process for the periINPlas than for the Dental water jet, which is based on an already CE-certified medical device. This explains lower number of results of safety testing presented in this article for the Dental water jet as compared to periINPlas.

The results of this study can be transferred to the clinical situation only to a limited extent. The in-vitro treatment was performed under optimal conditions, as access to the discs was unobstructed. This treatment is easier than in the patient's mouth. In addition, the flat discs do not correspond to the inherently complex thread morphology of dental implants. However, since all methods suffer from these limitations in clinical use, it is reasonable to assume that the method that performs best in-vitro will also offer advantages in the clinic. We would even argue that usability of WJ and CAP is better in the clinical setting as compared to CC due to the geometry of both handpieces which are well adapted for dental treatment.

## Conclusions

The newly developed water jet device effectively removes biofilm from sand-blasted, acid-etched titanium surfaces in-vitro and, in combination with the new cold atmospheric pressure plasma device, biologically acceptable surfaces allow osteoblasts to grow. Within the limits of the in-vitro study, WJ in combination with CAP results in cleaner surfaces in-vitro than the use of curette and cotton swabs with or without subsequent plasma treatment. Clinical healing of peri-implantitis lesions should therefore be better after treatment with the new combination therapy. A planned randomised clinical trial will provide clarity on the future clinical use of our new devices, which is registered under DRKS-ID: DRKS00026673) [59].

## Supplementary Information

**Additional file 1.** Permission to use the devices on patients.

**Additional file 2.** Images of the water contact angle measurement.

**Additional file 3.** Scanning electron micrograph of scratched titanium.

**Additional file 4.** Test controls of mechanical cleaning methods.

## Data Availability

The datasets used and/or analysed during the current study are available from the corresponding author on reasonable request. The datasets generated and/or analysed for regulatory approval, which were not explicitly discussed in this study, are partially not publicly available due international regulations.

## Abbreviations

AC: Alternating current
CAP: Cold atmospheric pressure plasma
CC: Mechanical treatment with curette and cotton swab
CI: Confidence intervals
CC + CAP: Combined treatment of curette and cotton swab with subsequent cold atmospheric pressure plasma treatment
DC: Direct current
NC: Negative control specimen
OD: Optical density
PBS: Phosphate-buffered saline
PC: Positive control specimen
PLC: Patient leakage current
PSS: Physiological saline solution
SD: Standard deviations
SEM: Scanning electron microscope
UV, UV-C: Ultra–violet radiation, radiation in range of 100–280 nm (UV-C)
WJ: Water jet
WJ + CAP: Combined treatment of water jet with subsequent cold atmospheric pressure plasma treatment
