# Supplementary material for: Efficiency of biofilm removal by combination of water jet and cold plasma: an in-vitro study
Source: BMC Oral Health. 2022 May 6;22:157. doi: 10.1186/s12903-022-02195-1 (PMC9074283; doi:10.1186/s12903-022-02195-1)
Supplement: Supplementary file 1 — Additional file 1. Permission to use the devices on patients. [file 12903_2022_2195_MOESM1_ESM.docx]

# Appendix 1


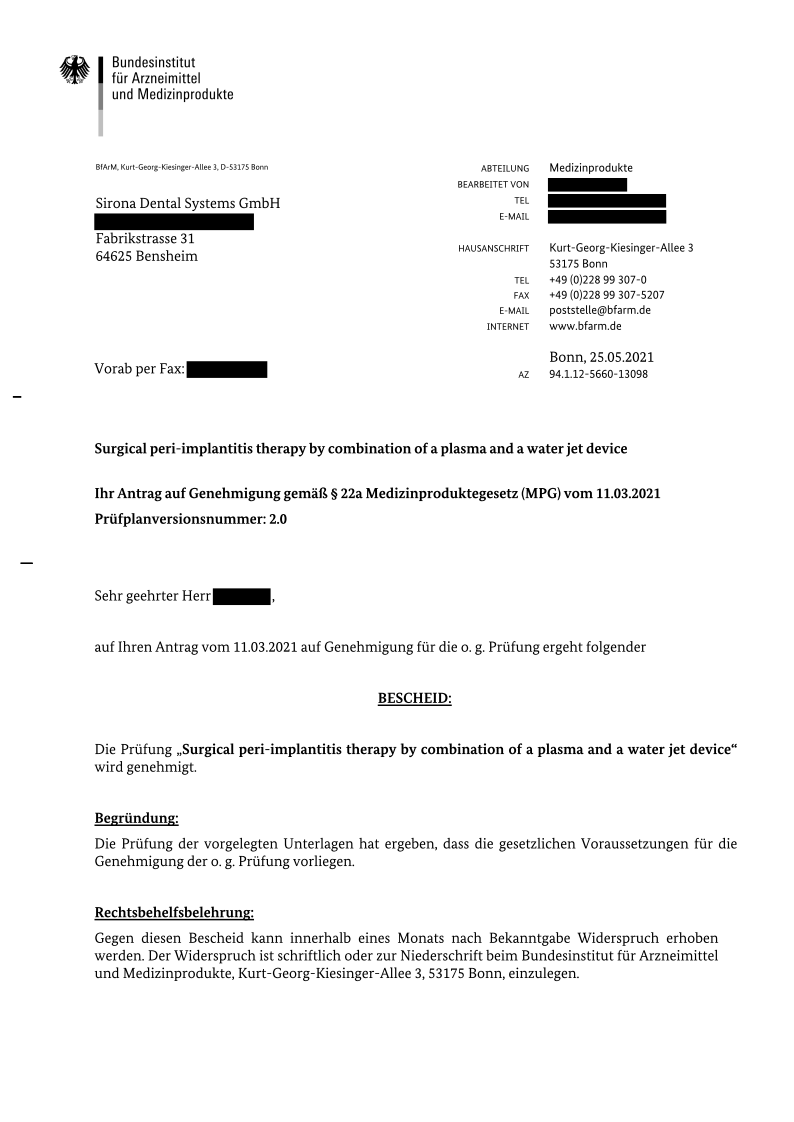


**Figure Appendix 1A:** Copy of the permission of committee of the German Federal Institute for Drugs and Medical devices.


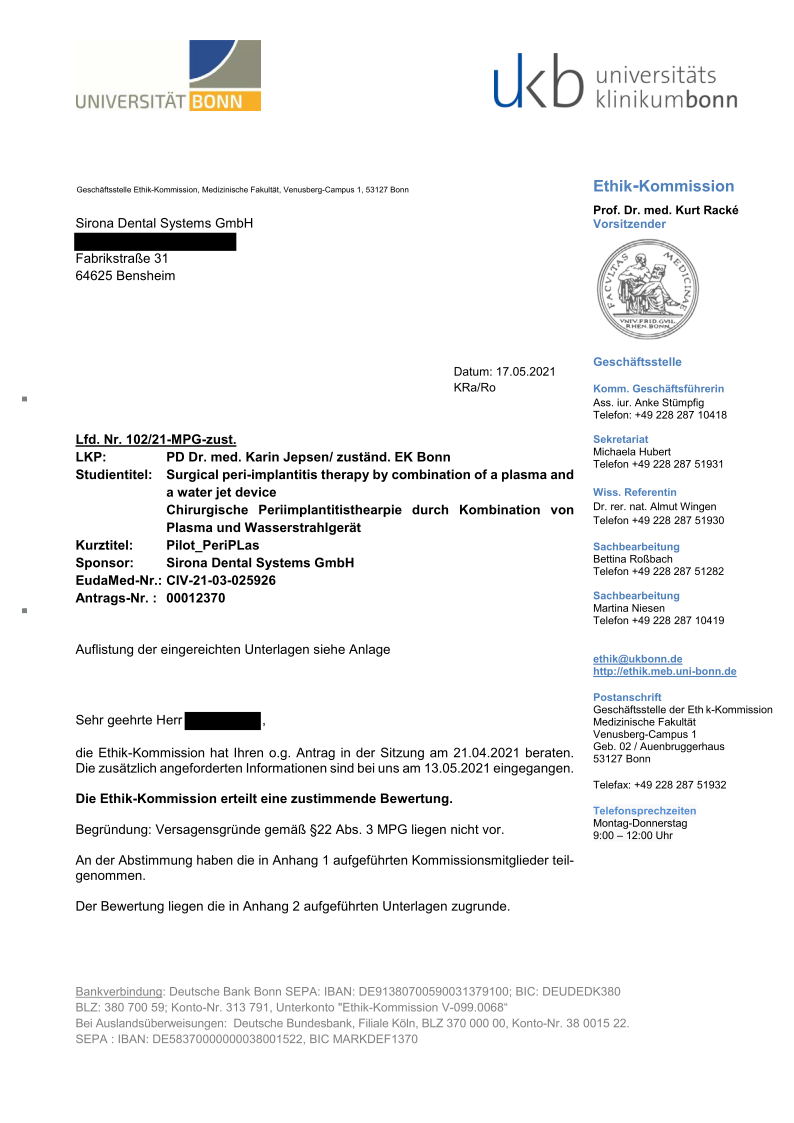


**Figure Appendix 1B:** Copy of the permission of ethic committee for the German Clinical Trials Register.
