# Supplementary material for: Efficiency of biofilm removal by combination of water jet and cold plasma: an in-vitro study
Source: BMC Oral Health. 2022 May 6;22:157. doi: 10.1186/s12903-022-02195-1 (PMC9074283; doi:10.1186/s12903-022-02195-1)
Supplement: Supplementary file 2 — Additional file 2. Images of the water contact angle measurement. [file 12903_2022_2195_MOESM2_ESM.docx]

# Appendix 2


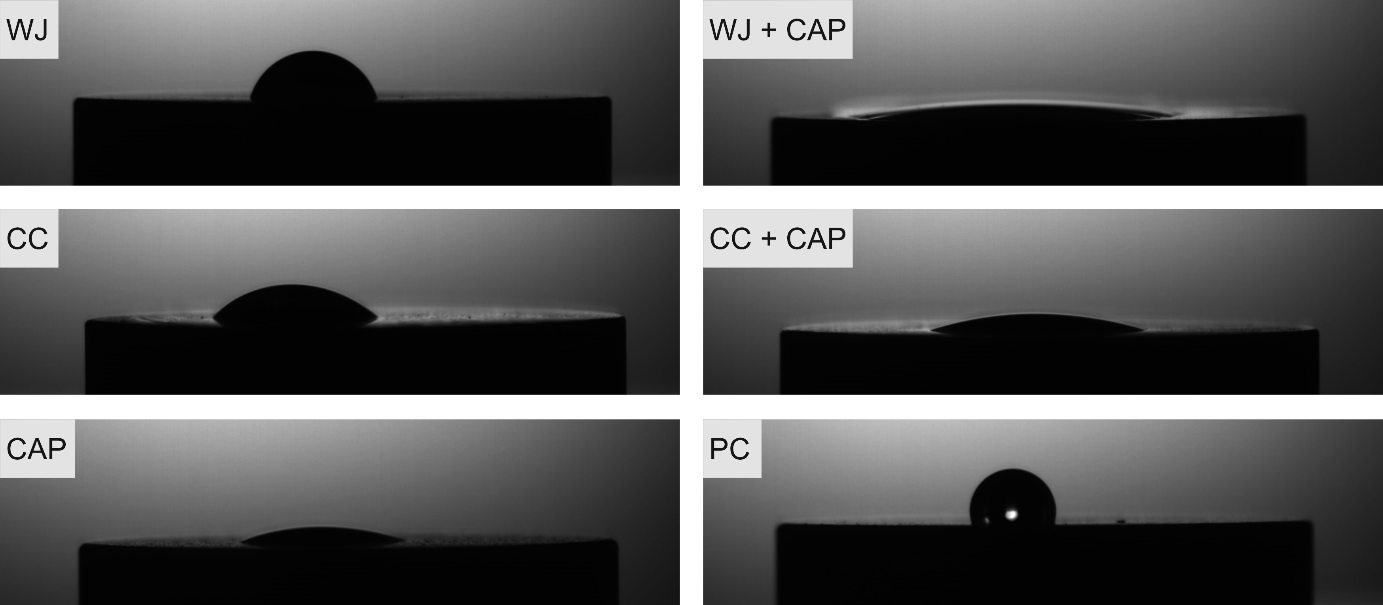


**Figure Appendix 2:** Images of water contact angle measurement after water drop on specimen treated by water jet (WJ), curette + cotton swab (CC), cold atmospheric plasma (CAP), combined treatment of WJ + CAP and CC + CAP, and the untreated positive control (PC). The time span between treatment and measurement was 2 h. The image of additionally immediately measurement after CAP (CAP*, time span 10 min) is missing.
