# Supplementary material for: Efficiency of biofilm removal by combination of water jet and cold plasma: an in-vitro study
Source: BMC Oral Health. 2022 May 6;22:157. doi: 10.1186/s12903-022-02195-1 (PMC9074283; doi:10.1186/s12903-022-02195-1)
Supplement: Supplementary file 3 — Additional file 3. Scanning electron micrograph of scratched titanium. [file 12903_2022_2195_MOESM3_ESM.docx]

# Appendix 3


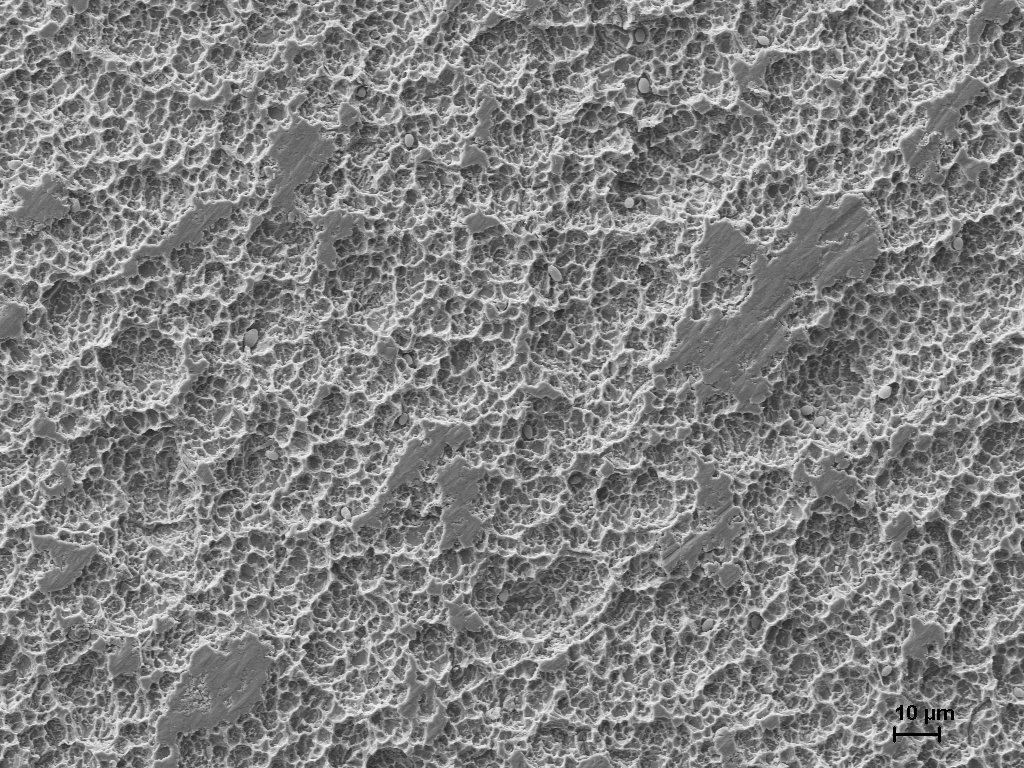


**Figure Appendix 3:** The scanning electron micrograph shows an example of a specimen with clear scratches that appeared after treatment with curette + cotton swab (CC). Scale bars: 10 µm.
