# Supplementary material for: Efficiency of biofilm removal by combination of water jet and cold plasma: an in-vitro study
Source: BMC Oral Health. 2022 May 6;22:157. doi: 10.1186/s12903-022-02195-1 (PMC9074283; doi:10.1186/s12903-022-02195-1)
Supplement: Supplementary file 4 — Additional file 4. Test controls of mechanical cleaning methods. [file 12903_2022_2195_MOESM4_ESM.docx]

***Appendix 4:*** *The two mechanical treatment methods, water jet, and curette + cotton swab were performed on sterile discs, to ensure that these treatment methods themselves were carried out sterile. The mean and Standard deviation (SD) based on OD values after reduced background noise (mean=0.06) for the time points after 0, 8, 16, 20, 24, 48, and 96 h.*

| **Time** | **WJ** | **CC** | **medium** |
| --- | --- | --- | --- |
|  | n=6 | n=5 | n=3 |
|  | **mean** | **mean** | **mean** |
|  | SD | SD | SD |
| 0 | **0.00** | **0.01** | **-0.01** |
|  | 0.00 | 0.02 | 0.00 |
| 8 | **0.00** | **0.01** | **0.00** |
|  | 0.00 | 0.01 | 0.00 |
| 16 | **0.00** | **0.01** | **0.00** |
|  | 0.00 | 0.02 | 0.01 |
| 20 | **0.00** | **0.01** | **0.00** |
|  | 0.00 | 0.01 | 0.01 |
| 24 | **0.00** | **0.00** | **0.00** |
|  | 0.01 | 0.01 | 0.01 |
| 48 | **0.00** | **0.00** | **0.00** |
|  | 0.00 | 0.01 | 0.00 |
| 96 | **0.00** | **0.01** | **0.00** |
|  | 0.00 | 0.01 | 0.00 |

*Abbreviations: WJ, water jet; CC, curette + cotton swab*
